# IMRT‐application with an add‐on MMLC

**DOI:** 10.1120/jacmp.v4i4.2498

**Published:** 2003-09-01

**Authors:** Thomas Tücking, Simeon Nill, Uwe Oelfke

**Affiliations:** ^1^ Department of Medical Physics Deutsches Krebsforschungszentrum Im Neuenheimer Feld 280 69120 Heidelberg Germany

**Keywords:** IMRT, connectivity, multileaf collimator, mini‐MLC

## Abstract

In order to provide automatic IMRT dose delivery with an add‐on MMLC a technical integration of a MMLC system with a linear accelerator was realized. The principle of this integration and the changes and enhancements of the existing hard‐and software are briefly described. The system was tested by the automatic delivery of an IMRT plan designed for a head and neck phantom. A verification of dose delivery was performed with film dosimetry. The plan consisting of 78 “step and shoot” segments could be delivered within 17 minutes. A high spatial accuracy of the fluence pattern at the isocentre was reached by a resolution of 2.75×2.75 mm^2^. The measured dose profiles were within 3% of the maximum dose of the calculated profiles.

PACS number(s): 87.53.–j, 87.90.+y

## I. INTRODUCTION

It is well known that the leaf width of a multileaf collimator (MLC) can be crucial for the quality of a treatment plan for irregular shaped target volumes.^1^ ^–^ ^3^ By comparative treatment planning for clinical cases optimized with different MLCs it has been shown that more conformal dose distributions for the target volume and a better sparing of adjacent critical structures could be achieved by using a miniature multileaf collimator (MMLC) instead of the linear accelerator's (linac) internal standard MLC.^4^

This paper reports the technical integration of the Moduleaf™ (MRC Systems, Heidelberg, Germany) add‐on MLC^5^ (a MMLC^a^ specifically designed for IMRT) with a Primus™ (Siemens OCS, Concord, CA) linac, to provide the option for automatic delivery of “step & shoot” IMRT sequences with an add‐on MMLC. An application and dosimetric verification of this dose delivery hardware system was performed.

## II. MATERIALS AND METHODS

### A. The flow of information

**Figure 1:**
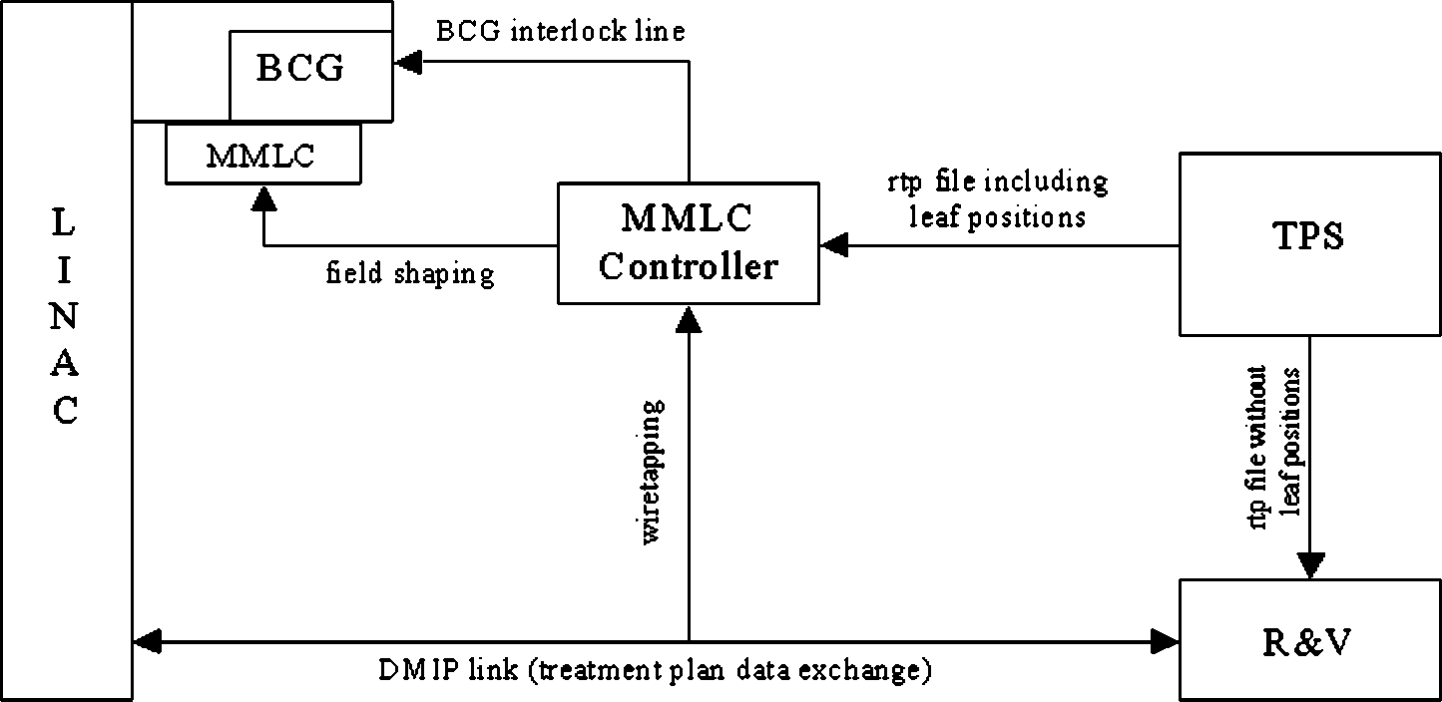
Schematic view of the information flow for the hardware integration of MMLC and linac. The interaction of the single components of the system is illustrated.

The integrated system of the MMLC with the linac must allow an automatic dose delivery process of the complete IMRT sequence within a clinically acceptable delivery time. For this purpose a synchronization of the information transfer between those two usually independently working systems has to be assured (Fig. 1). The starting point of the information flow is the treatment planning system (TPS), which generates two radiotherapy plan (RTP) files instead of only one for IMRT treatments without the add‐on MMLC. Those files contain all parameters required for the delivery of the IMRT treatment sequence like gantry angle, collimator angle, energy, etc. for each “step & shoot” segment of the dose delivery process. The files are transferred to the linac's Record & Verification System (R&V) and to the MMLC controller. The RTP file transmitted to the R&V does not contain any MLC leaf position but only the jaw positions to create a rectangular field of the primary radiation beam for each IMRT segment. The other RTP file, which is transmitted to the MMLC controller, includes all MMLC leaf positions for each segment. Furthermore, in both RTP files each segment of the IMRT sequence is labeled by an additional accessory code that uniquely identifies that segment within the treatment plan in both RTP files.

The R&V and the linac console communicate via the Digital MEVATRON Interface Protocol^b^ (DMIP) link (see Fig. 1). At the beginning of the treatment and before each new segment, the R&V sends the required treatment parameters including the additional accessory code of the current segment via the DMIP link to the linac console. When the delivery of a segment is finished the linac console informs the R&V about this fact, and the next segment parameters are uploaded to the linac console by the R&V This is repeated until the last segment has been transferred. The MMLC controller is able to listen to the data flow exchanged between the R&V and the linac console via an additional link (see Fig. 1).

With the data received from the DMIP link, especially with the accessory code for the current segment, the MMLC controller can identify which IMRT treatment segment will be executed next by the linac. The MMLC controller can then load the leaf positions for the selected segment from its RTP file. For verification purposes all other segment parameters like patient ID, geometric parameters, etc. are compared with those received from the DMIP link. If the settings are confirmed, the leaves are driven to their prescribed positions and the MMLC controller removes the block code generator (BCG) interlock (see Sec. IIB) to unblock the current segment for delivery.

### B. The BCG as the MMLC‐linac interface

A BCG was developed and installed inside the linac's gantry. This additional hardware component represents an electronic interface between the MMLC controller and the linac to communicate the current MMLC state to the linac console. The BCG allows the MMLC controller to block the radiation beam as long as the MMLC leaves have not yet reached their positions required for the delivery of the current IMRT segment. Therefore, the MMLC controller transfers the accessory codes to the BCG where they are transformed into hardware states at the accessory interface of the linac. These states correspond to the additional accessory codes for each segment within the RTP files. The linac continuously checks all hardware parameters by the R&V for the current segment. This includes the comparison of the additional accessory code labeling the segment with the code presented by the BCG. The BCG only provides the corresponding accessory code for a treatment segment if the MMLC controller has verified all leaf positions. Therefore, the irradiation for a segment cannot be started before the correct field shape is set by the add‐on MMLC.

One special hardware state of the BCG corresponds to a “not ready” accessory code, which cannot be used to identify a treatment segment. Consequently the MMLC controller can block the radiation at any time by presenting the “not ready” code via the BCG. This is done if the MMLC leaves have not reached the targeted positions of the current IMRT segment or if an error such as a leaf position falling out of tolerance occurs within the MMLC system during a beam‐on phase. If the BCG is powered off it presents no accessory code at all. This operational mode is used for treatments without an add‐on MMLC. A failure of the BCG immediately interrupts the treatment because a missing accessory code is interpreted as a mismatch of the required hardware parameters. Furthermore, an additional interlock at the linac is set by the MMLC controller whenever any hardware component is not working properly. The MMLC‐ and the linac controller store the status of an interrupted treatment so that it can be resumed later.

The BCG was technically realized by resistors that can be switched selectively via relays that are controlled by input/output data lines of the MMLC controller. The linac system recognizes the resistor combinations and the linac console interprets them as accessory codes. The MMLC controller can verify the resistor values presented by the BCG and notify the operator if the BCG is not functioning properly.

### C. The automatic dose delivery process

A treatment starts with the BCG presenting the “not ready” code. After the MMLC controller has verified the leaf positions of the MMLC for the first segment the BCG gets the signal to present the corresponding accessory code. When the linac console has accepted the BCG's new code as the expected accessory code for the IMRT segment the linac will start the treatment. If the segment has been completely delivered the linac console sends a status signal to the R&V via the DMIP link. Initiated by this signal the MMLC controller presents the “not ready” code via the BCG and waits for the next segment, which is determined by the data sent via the DMIP link from the R&V to the linac console.

In this way the complete delivery of the IMRT plan is done automatically. As the IMRT treatment mode with the standard internal MLC the linac gradually processes the IMRT sequence and in addition the add‐on MMLC is modulating the fluence with its own leaves.

### D. Tests and verification of dose delivery

**Figure 2:**
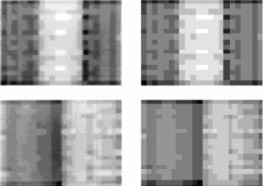
Two gray tone distributions measured by film at the exit plane of the MMLC (left) and corresponding fluence distributions calculated by the TPS (right). The fluence distributions are generated by 17 (upper distributions) and 16 (lower distributions) segments.

**Figure 3:**
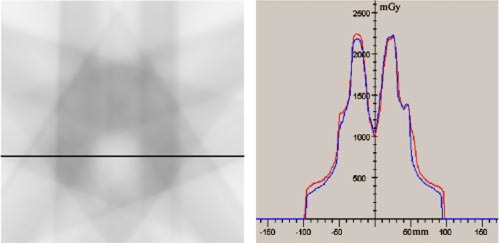
(Color) The film on the left shows a 10×10 cm^2^ area cut‐out of the measured dose distribution in the isocentric slice of the head and neck phantom. On the right‐hand side a measured line dose profile (red) compared to the corresponding calculated dose profile (blue) is shown. Both are taken along the solid black line on the film (left panel).

Automatic delivery of an IMRT plan designed for a horseshoe shaped target volume that winds around a critical structure in analogy to a typical paraspinal tumor was tested with a prototype of the developed integrated system of the Primus™ linac and the ModuLeaf™. For treatment planning the planning systems VIRTUOS^6^ and KonRad^7^ for the inverse planning were used. The plan consisted of five coplanar, equally spaced beams divided into 78 segments. A verification process was performed for the fluence distributions of each of the five beams and for the dose distribution in a phantom for the composite plan. To qualitatively verify the fluence distributions of the single beams, a film at the exit plane of the MMLC was irradiated with the associated segments (Fig. 2). These films were visually compared to the calculated fluence maps assuming that the relative gray tones on the film show a first order approximation of the fluence values. For the verification of the composite treatment plan film dosimetry inside an in house constructed head and neck phantom was applied according to a published technique.^8^ Five Kodak EDR2 films were placed into transverse slices of the phantom with a spatial separation of 1 cm whereby the central film was placed into the isocentric slice (Fig. 3). The whole sequence was delivered to the phantom and the measured dose distributions were compared to the calculated distributions. Several profiles of the measured dose distributions for each film were compared to the corresponding profiles taken from the 3D dose cube provided by the TPS.

## III. RESULTS AND DISCUSSION

A 2.75×2.75 mm^2^ resolution of the fluence map in the isocenter plane could be attained with the add‐on MMLC which can be quite useful for complexly shaped tumor cases in the head and neck region. The gray tone distributions of the films irradiated with the five single beams, each consisting of up to 18 irradiation segments, showed a good correlation to the prescribed fluence patterns. All patterns and single bixels of (2.75 mm)^2^ from the calculated fluence maps could be identified on the films (Fig. 2). The high spatial resolution of the fluence could be recognized on the films. This indicates that the IMRT delivery with the used integrated system is feasible with a high accuracy.

The results of the dosimetrical evaluation of the films for the composite plan showed no significant deviation between the measured dose distribution and the 3D dose distribution calculated by the TPS. All measured dose profiles were within 3% of the maximum dose of the calculated profiles.

The complete IMRT sequence of 78 “step and shoot” segments could be delivered within 17 min so that the average time for the delivery of a single segment was about 13 sec, which is clinically acceptable. The average delivery time per segment is only slightly increased compared to the one for the internal MLC of this linac. The prototype of the integrated system of MMLC and linac with the hard‐ and software modules was very reliable in our tests. No technical problems or failures occurred during the extensive testing period. Since the IMRT delivery is performed automatically the only additional steps for the operator is to start the MMLC control software before the beginning of the treatment. All subsequent steps need only to be done at the R&V console. The corresponding actions at the MMLC are performed automatically.

## IV. CONCLUSIONS

A system for automatic delivery of “step & shoot” IMRT sequences with an add‐on MMLC was developed and tested. A main component is the BCG, a hardware interface installed inside the linac. With this component the MMLC system has the possibility to present an additional accessory code to the linac that can enable or block the radiation beam at any time. This hardware and the data received from the DMIP link enable the MMLC controller to synchronize both systems (linac and MMLC), which is fundamental for the automatic process of an IMRT treatment sequence.

The reported evaluation of the considered hardware configuration shows that an automatic IMRT dose delivery with an add‐on MMLC is feasible. Moreover, it was demonstrated that the achievable dose distributions showed the expected high spatial resolution and therefore provides an enhanced clinical potential for a specific class of IMRT treatments. Our investigations are the basis of further developments, tests, and validations aiming to bring this system into clinical routine in the near future.

## ACKNOWLEDGMENTS

We would like to thank P. Häring and B. Rhein for their assistance during the measurements. We would also like to thank S. Seeber and J. Juschka from MRC Systems for their support during the development.

## Footnotes

1 Technical specification of the ModuLeaf™: 40 leaf pairs, 2.75 mm leaf width at isocenter (SCD: 61.3 cm), maximum field size at isocenter: 13.2×11.0 cm, maximum leaf travel over central axis: 6 cm.

2 Digital MEVATRON Interface Protocol is a trademark of Siemens Medical Solutions USA, Inc.
